# Temporal and Spatial Patterns of Habitat Use by Juveniles of a Small Coastal Shark (*Mustelus lenticulatus*) in an Estuarine Nursery

**DOI:** 10.1371/journal.pone.0057021

**Published:** 2013-02-20

**Authors:** Malcolm P. Francis

**Affiliations:** National Institute of Water and Atmospheric Research Ltd, Wellington, New Zealand; Macquarie University, Australia

## Abstract

Juvenile rig (*Mustelus lenticulatus*) were internally tagged with acoustic transmitters and tracked with acoustic receivers deployed throughout two arms of Porirua Harbour, a small (7 km^2^) estuary in New Zealand. Ten rig were tracked for up to four months during summer–autumn to determine their spatial and temporal use of the habitat. The overall goal was to estimate the size of Marine Protected Areas required to protect rig nursery areas from direct human impacts. Rig showed clear site preferences, but those preferences varied among rig and over time. They spent most of their time in large basins and on shallow sand and mud flats around the margins, and avoided deep channels. Habitat range increased during autumn for many of the rig. Only one shark spent time in both harbour arms, indicating that there was little movement between the two. Rig home ranges were 2–7 km^2^, suggesting that an effective MPA would need to cover the entire Porirua Harbour. They moved to outer harbour sites following some high river flow rates, and most left the harbour permanently during or soon after a river spike, suggesting that they were avoiding low salinity water. Rig showed strong diel movements during summer, although the diel pattern weakened in autumn. Persistent use of the same day and night sites indicates that diel movements are directed rather than random. Further research is required to determine the sizes of rig home ranges in larger harbours where nursery habitat is more extensive. Marine Protected Areas do not control land-based impacts such as accelerated sedimentation and heavy metal pollution, so integration of marine and terrestrial management tools across a range of government agencies is essential to fully protect nursery areas.

## Introduction

The juvenile stages of marine fishes often inhabit distinct areas, frequently called nurseries, where they are spatially separated from older fish of the same species. Estuaries, shallow harbours and coastal waters are extensively used by inshore fish species as nursery grounds. These habitats are often flanked by high-density human populations and have been increasingly subjected to human modification and degradation. They may be impacted by commercial and recreational fishing, sedimentation, eutrophication, pollution, dredging, marina development and reclamation. Loss or degradation of nursery grounds, or high mortality of juveniles on these grounds, could have serious consequences for the sustainability of fisheries for these species, and for the health of the ecosystem.

Until recently, the term nursery was often used loosely to include any area in which juveniles occurred. Beck et al. [1] reviewed existing definitions and proposed a more rigorous one: a nursery is a region where juvenile fish occur at higher densities, avoid predation more successfully and grow at a faster rate, thereby providing a greater relative contribution to adult recruitment than other areas. This means that only areas that contribute proportionally more to the adult stock than average can be considered nurseries [2]. This definition is difficult to apply, because measuring the spatial success of recruitment to an adult population is rarely possible. Heupel et al. [2] developed a new nursery definition for sharks as follows: “Three criteria [must be] met for an area to be identified as a nursery: (1) sharks are more commonly encountered in the area than in other areas; (2) sharks have a tendency to remain or return for extended periods; (3) the area or habitat is repeatedly used across years”. These criteria are much easier to apply than the more general definition of Beck et al. [1].

Most elasmobranchs are born or hatch at a large size, often 20–40 cm total length (TL). They therefore bypass the highly vulnerable planktonic egg and larval stages of most teleost fishes, and as a result experience greatly reduced natural mortality rates. A consequence of this is that there is probably a close relationship between stock size and recruitment in elasmobranchs; i.e. large populations of adults translate directly into high levels of recruitment, and small adult populations produce few recruits. In technical terms, the stock-recruit steepness parameter is low compared with those of most teleosts. Because of their low fecundity and often lengthy reproductive cycles, elasmobranchs have little capacity to compensate for increased juvenile mortality, which means that healthy and viable nurseries are vital for maintaining adult population sizes.

In New Zealand, protection of habitats of particular significance for fisheries management is an environmental principle of the Fisheries Act 1996, and the Minister for Primary Industries is required to take these habitats into account when managing fisheries. Furthermore, the National Plan of Action–Sharks (NPOA–Sharks), which was approved in October 2008, states that “a range of actions will be implemented to ensure that fisheries management in New Zealand satisfies the objectives of the International Plan of Action–Sharks to ensure the conservation and management of sharks and their long-term sustainable use” [3]. The NPOA–Sharks identified the following important action: “identification of areas of habitat of particular significance to shark species (e.g. spawning, pupping and nursery grounds)”.

Once identified, elasmobranch nurseries may require a multi-pronged management approach to protect them and their inhabitants. Spatial management tools such as Marine Protected Areas (MPAs) can confer protection against a range of human impacts, such as fishing and physical destruction and degradation of habitat (e.g. by dredging, reclamation, or construction of marinas). Tracking studies have shown that a number of small coastal shark species exhibit strong site fidelity and have restricted home ranges that often increase with age and size [4]–[11]. MPAs are likely to confer greater benefits to species or life history stages that remain within MPA boundaries for a longer time, particularly during periods of high anthropogenic mortality [6], [9], [12].

Other impacts of terrestrial origin such as sedimentation and heavy metal pollution are more of a challenge, and may require large-scale changes in the way adjacent cities and rural communities operate. Thus, conservation of elasmobranch nurseries may involve multiple central and regional government authorities, as well as a wide range of sector and community groups. Support and “buy-in” by the public is likely to be crucial for successful nursery protection.

In order to manage nurseries, we first need to locate them, and then understand how fish use them in time and space [12]. These key information requirements underpin the design and implementation of an appropriate suite of management tools and actions.

Not all inshore elasmobranchs have discrete nurseries [13]. However, the coastal waters of New Zealand appear to be used as nursery grounds by juveniles of several elasmobranchs including two small sharks (rig *Mustelus lenticulatus* and school shark *Galeorhinus galeus*), three rays (long-tailed stingray *Dasyatis thetidis*, short-tailed stingray *D. brevicaudata*, and eagle ray *Myliobatis tenuicaudatus*), and a chimaeroid (elephantfish *Callorhinchus milii*). The present study aims to understand how rig, also known as smooth or spotted dogfish, uses estuaries as a nursery ground. Rig are small endemic sharks that reach a maximum length of 1.51 m TL; they support a small commercial fishery catching 1,200 tonnes per year, and a small recreational fishery [14]. During spring, adult rig migrate from the continental shelf into shallow coastal waters where the females give birth to live young 25–30 cm long, mate with the males, and then return to deeper water [15]–[18]. The juveniles inhabit estuaries in summer–autumn and then depart for the open sea at a length of about 50 cm [16]. However, little is known about the parts of estuaries that juveniles use, whether this changes with time, how long they remain in estuaries, and what physical or biological features of estuaries are important to them. An overall goal of this study is to estimate what size MPA would be required to protect rig nursery areas, and this requires definition of the spatial scale of rig habitat use. To address these gaps in our knowledge, juvenile rig were tracked with acoustic tags for up to four months during summer–autumn in a medium-sized estuary in southern North Island, New Zealand.

## Methods

### Ethics statement

Fishing operations were conducted under a Special Permit from the New Zealand Ministry of Fisheries (now the Ministry for Primary Industries). Rig tagging was carried out under NIWA Animal Ethics Committee approval number 107.

### Study area

**Figure 1:**
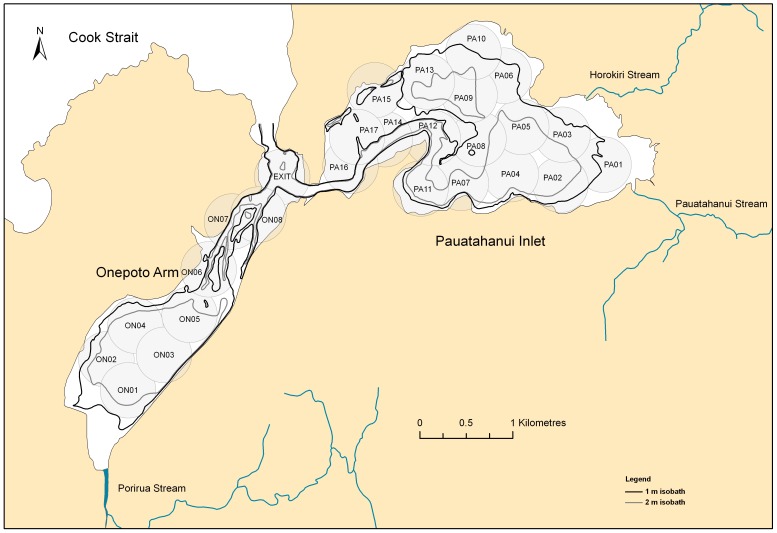
Map of Porirua Harbour showing Onepoto Arm and Pauatahanui Inlet and the channels linking them. Also shown are the 1 m and 2 m depth contours (below mean sea level) and the locations and approximate detection ranges (300 m radius grey circles) of the 26 acoustic receivers.

Porirua Harbour is a tidal estuary situated on the fringes of Porirua City (ca 50,000 people), near Wellington, New Zealand (Figure 1). Its catchment consists of a mixture of city and urban development (14% of area), grassland (46%), and forest and shrubs (37%) [19]. The harbour comprises two distinct parts, Pauatahanui Inlet and Onepoto Arm, that are linked by a channel that connects them to the open coast at the northern entrance to Cook Strait, which separates North and South islands of New Zealand.

Both of the harbour arms are 3.5 km long, but Pauatahanui is wider (maximum width 2.0 km) than Onepoto (1.0 km). Pauatahanui covers about twice the area of Onepoto (4.7 km^2^ and 2.4 km^2^ respectively [20]), and Porirua Harbour has an overall area of 7 km^2^. The harbour is very shallow with extensive intertidal flats and banks of muddy sand that are exposed at low tide. Each arm of the harbour has a central basin varying from 0.5 m to 2.5 m deep (below mean sea level) [21], [22]. These basins were deeper historically, and have been infilling as a result of accelerated terrestrial erosion. Between 1974 and 2009, the net average deposition rate was 5.7 mm per year in Onepoto Arm and 9.1 mm per year in Pauatahanui Inlet [20]. Tidal flows of more than 3 knots have scoured out channels at the entrances to both harbour arms [23]. These channels are narrow, steep-sided and lined with shells, pebbles and coarse sand. The channels reach depths of 3–5 m and 5–8 m near the entrances of Onepoto and Pauatahanui respectively, and a hole 12–18 m deep has been scoured out where the outflows from the two arms merge [21]–[23].

The Porirua Harbour catchment receives an annual rainfall of about 1,200 mm, and many small streams enter the harbour. The main inflows come from Porirua Stream at the head of Onepoto, and Horokiri and Pauatahanui streams at the eastern end of Pauatahanui (Figure 1) [19]. Rain runoff reduces the salinity of the harbour, particularly at the head of Pauatahanui [24] and presumably also Onepoto. Salinity stratification of the water column occurs, particularly during winter and spring, with low salinity water at the surface and high salinity water near the seabed [24]. At the entrance to Porirua Harbour, the maximum spring tide range is about 1.3 m. The harbour does not drain completely, with 65% of its area remaining covered at low tide [19].

### Acoustic tracking

**Figure 2:**
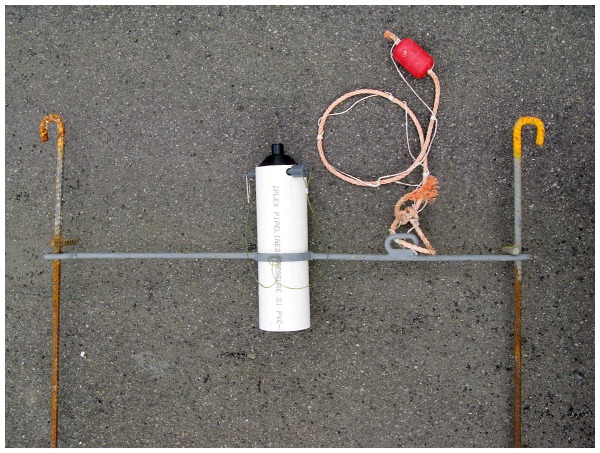
Photo of acoustic receiver assembly before deployment. The VR2W receiver is mounted inside the white PVC tube with its transducer exposed and pointing upwards. The PVC tube is buried in the substratum to the level of the horizontal metal yoke, which is anchored to the seabed with two 90 cm long metal stakes. A small float is attached to the yoke to assist divers in finding the receiver. The curved hooks on the tops of the stakes facilitate recovery of lost receivers by snagging a rope dragged along the seabed.

Vemco coded acoustic transmitters (tags) were used in conjunction with VR2W acoustic receivers to track juvenile rig. These tags emit unique trains of sound pings, which are then decoded and recorded (along with a date-time stamp) by any receivers within range. Receivers were mounted inside a PVC pipe and half-buried in the substratum by a diver (Figure 2). The transducer projected about 15 cm above the substratum. At the end of the experiment, the whole assembly was recovered by a diver, who located them using GPS co-ordinates.

A range test carried out with two receivers showed that detection success declined approximately linearly from 78–82% of pings at a range of 10 m to 18–30% at 300 m and 0–2% at 500 m. This suggested that the receivers would probably detect rig at ranges up to about 300 m, so an array of receiver sites spaced approximately 600 m apart was deployed (Figure 1). This resulted in an almost complete overlapping coverage of receiver detection ranges in both arms of the harbour, except for some of the intertidal sand flats that drain at low tide. Acoustic receivers were deployed on 21–28 January 2009 and recovered five months later on 23–24 June 2009. The receiver situated in the channel at the junction between the Onepoto and Pauatahanui arms was designated the EXIT site, because all rig had to pass this receiver to reach the open sea (Figure 1).

Juvenile rig were caught on 29–30 January 2009 with 50 mm-mesh monofilament nylon nets set for less than 30 minutes. Lively, healthy sharks were measured, sexed and placed in a large plastic bin that had been filled with seawater from the capture site to maintain the rig at ambient conditions. Pure oxygen was bubbled through the water.

Cylindrical Vemco V9-2L tags that were 9 mm in diameter, 29 mm long, and weighed 4.7 g (2.9 g in water) were programmed to emit sound pings at 30–90 s intervals. Tags were surgically inserted into the rear of the body cavity. The aim was to limit tag weight to 2% or less of body weight, giving a minimum rig weight of 235 g which corresponds with a minimum TL of about 38 cm. Rig were inverted to induce tonic immobility, and supported with a submerged foam cushion with the head and gills immersed in oxygenated seawater and the posterior, ventral surface of the body in air. A small incision was made in front of the pelvic fins and to one side of the midline, the tag was inserted, and the incision was closed with one silk suture. Surgical instruments and tags were soaked in Betadine antiseptic solution prior to use. Tagged rig were held in oxygenated seawater for a few minutes to observe their recovery from the procedure, and then released into the sea near where they were caught.

Date-time stamps were recorded in universal time (UTC). Analyses involving time of day were converted to New Zealand Standard Time by adding 12 hours.

### Environmental data

As a proxy for the amount of rain runoff entering Porirua Harbour, daily river flow rates were obtained from a river flow gauge at Pauatahanui Gorge site, about 2 km up Pauatahanui Stream from Pauatahanui Inlet (ca 41.117°S, 174.923°E) (National Institute of Water and Atmospheric Research, unpubl. data). Sea surface temperature (SST) data recorded at Mana Marina adjacent to the EXIT receiver site (Figure 1) were obtained from 14 February to the end of May 2009, thus covering all but the first two weeks of the acoustic tracking experiment (Greater Wellington Regional Council, published data).

### Data analysis

Descriptive analyses of rig movements were based on acoustic tag detections at individual receivers. Data were expressed as percentages of the detections received, and were not analysed statistically because such data are pseudoreplicated. A Residency Index, defined as the number of days a tag was detected by the array divided by the number of days the rig was present in Porirua Harbour [6], [11], [25], was calculated for each rig to determine its residence period and the monitoring effectiveness of the array. A niche overlap index was calculated to determine the degree to which rig used the same ‘resource’, defined here as the detection radius around a single acoustic receiver. Similar studies have often used the Pianka Index [10], [26] but that index has performed poorly in simulations [27]. Instead the Proportional Similarity (PS) Index, also known as Czekanowski Index, was used [28]. This index, which ranges from 0 for no overlap to 1 for complete overlap, can give biased results with small sample sizes and high overlap [29], [30], so analyses were restricted to the seven rig having track lengths of 89 days or more. Analyses were carried out in EcoSim 7.71 [31], and PS Index significance was assessed by running 1000 simulations using the RA3 algorithm [26], [31].

To determine whether rig distribution was affected by freshwater runoff into the estuary, the distance of each receiver from the nearest of the three main streams (two in Pauatahanui and one in Onepoto, see Figure 1) was calculated. For each rig, the mean daily distance from the nearest river was calculated by averaging the receiver distances for all its detections across each day. Pearson correlation coefficients were calculated between the mean daily distance and the daily flow rates of Pauatahanui Stream for eight rig having track lengths of 27 days or longer. A Dunn-Sidak adjusted significance level of p = 0.0064 (k = 8 comparisons, [32]) was used to maintain the experimentwise error rate at p = 0.05. Correlation coefficients were also calculated using distance lags of 1 to 6 days.

## Results

Fifteen juvenile rig were tagged and released, nine in Pauatahanui and six in Onepoto (Table 1). Based on its length of 58 cm TL, the largest rig (rig 52763) was judged to have been in its second year (1+ year class) [16], [33]. The remaining 14 tagged rig were juveniles (0+ age class) 34–42 cm long (mean 38.8 cm). Four of the tagged rig were shorter than the target minimum length of 38 cm, but this is not thought to have been a problem as transmitter weight to body weight ratios of 3–12% have been found to cause no observable deleterious effects in other fishes [34]. Females outnumbered males 11∶4 (Table 1).

**Table 1:** Tag release details for 15 *Mustelus lenticulatus*, their subsequent fate (whether they died or departed from the harbour), and the length of the acoustic detection record (time between release and death or departure from Porirua Harbour).

Date tagged in 2009	Harbour arm	Tag number	Total length (cm)	Sex	Fate	Last live detection	Time to death or departure (days)
29 January	Pauatahanui	52751	38	Male	Departed	30 April	91
29 January	Pauatahanui	52752	38	Female	Departed	29 April	90
29 January	Pauatahanui	52753	40	Male	Departed	23 May	114
29 January	Pauatahanui	52754	37	Female	Departed	28 April	89
29 January	Pauatahanui	52755	41	Female	Died	5 February	7
29 January	Pauatahanui	52756	37	Female	Died	2 February	4
29 January	Pauatahanui	52757	36	Female	Departed	25 February	27
29 January	Pauatahanui	52758	41	Female	Departed	9 May	100
29 January	Pauatahanui	52759	40	Male	Departed	8 February	10
30 January	Onepoto	52760	42	Female	Departed	29 April	89
30 January	Onepoto	52761	41	Male	Died	7 February	8
30 January	Onepoto	52762	39	Female	Departed	6 May	96
30 January	Onepoto	52763	58	Female	Departed	12 February	13
30 January	Onepoto	52764	39	Female	Died	23 February	24
30 January	Onepoto	52765	34	Female	Died	21 February	22

All 26 receivers were successfully recovered and downloaded. Three receivers in deep channels (sites PA17, ON08, and EXIT, see Figure 1) were buried by coarse sand when recovered, but all were still recording tags until near the end of the experiment. Detections from five tags were initially recorded from a number of sites, and then after 4–24 days changed to a pattern of detections coming mainly from a single site, with occasional detections from 1–3 adjacent sites. I interpret this detection pattern as a cessation of movement of the tag, most likely resulting from death of the rig, although expulsion of the tag through the surgical wound is also possible. Three of these rig died within about one week of release (4–8 days) and the other two died after about three weeks (22–24 days) (Table 1). Death may have resulted from stress or injury caused by set net capture, trauma from the surgical procedure and tagging, infection, or a combination of these.

The remaining 10 rig were detected at multiple sites throughout a harbour arm during their period of residence (see below), and they provided acoustic records for 10–114 days (mean 72 days) (Table 1). These rig appear to have been healthy and active, and are assumed to have been behaving normally. Data from the first 2–3 days post-tagging (up to 31 January) were discarded to remove any short-term tagging effects on behaviour. The reduced data set for 10 healthy rig (seven in Pauatahanui and three in Onepoto; seven females and three males) consisted of 375,227 detections between 1 February and 23 May 2009. This reduced dataset was used for all subsequent analyses.

All rig were recorded by at least one receiver on every day before their departure from the harbour (Residency Index = 1), indicating that the array was effective in monitoring rig presence in the harbour, and that all rig remained within Porirua Harbour for the duration of their track. All but two of these rig were detected at the EXIT site on the last day of their record, indicating that they had left Porirua Harbour. The other two rig (52757 and 52759) were detected at site PA16 the day before and the day of their last record respectively, and likely departed the harbour without being detected by the EXIT receiver. The last records for departing rig were dated between 8 February and 23 May (Table 1).

**Figure 3:**
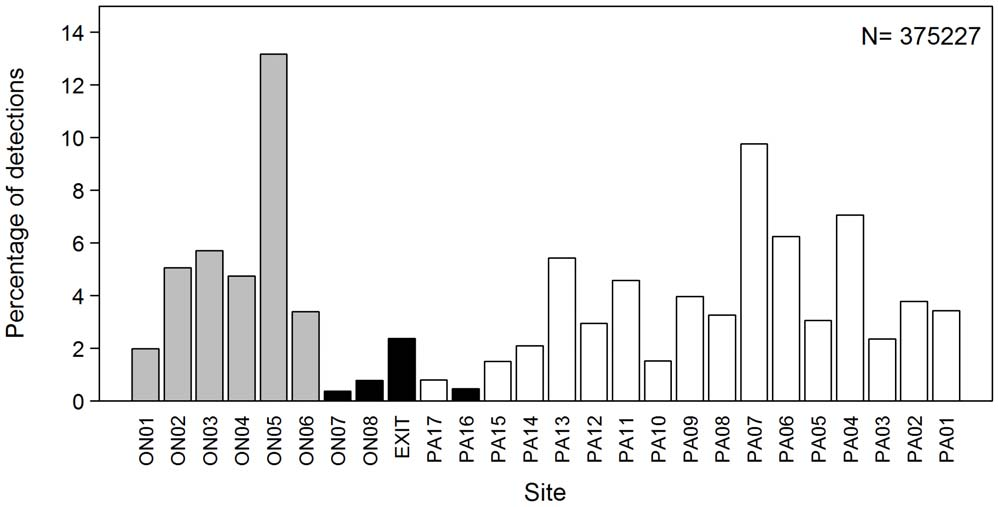
Frequency distribution of acoustic tag detections by site, all rig combined. Receiver sites are shown in Figure 1 and are grouped by Onepoto Arm (grey bars), Pauatahanui Inlet (white), and sites in both arms that have deep channels (black). N, sample size.

Tagged rig were detected at all 26 sites, though the distribution of detections was highly uneven. Large numbers of detections were recorded throughout the shallow inner and central parts of both harbour arms, particularly at the northern end of the Onepoto basin (site ON05), the southern Pauatahanui basin (PA04 and PA07) and the northern shallow part of Pauatahanui (PA06) (Figure 3). Sites near the harbour entrances that were dominated by deep channels (ON07, ON08, EXIT, PA17 and PA16) all had low numbers of detections.

For five rig tagged in Pauatahanui and having tracks of 89 days or longer, the PS Index ranged from 0.61 to 0.82 (mean = 0.71) for the ten pairwise comparisons. The PS Index was 0.95 for the single pairwise comparison between two rig tagged in Onepoto and having tracks of 89 days or longer. Associations between pairs of rig within harbour arms were all highly significant (p≤0.001), indicating overlapping habitat use at the daily level.

**Figure 4:**
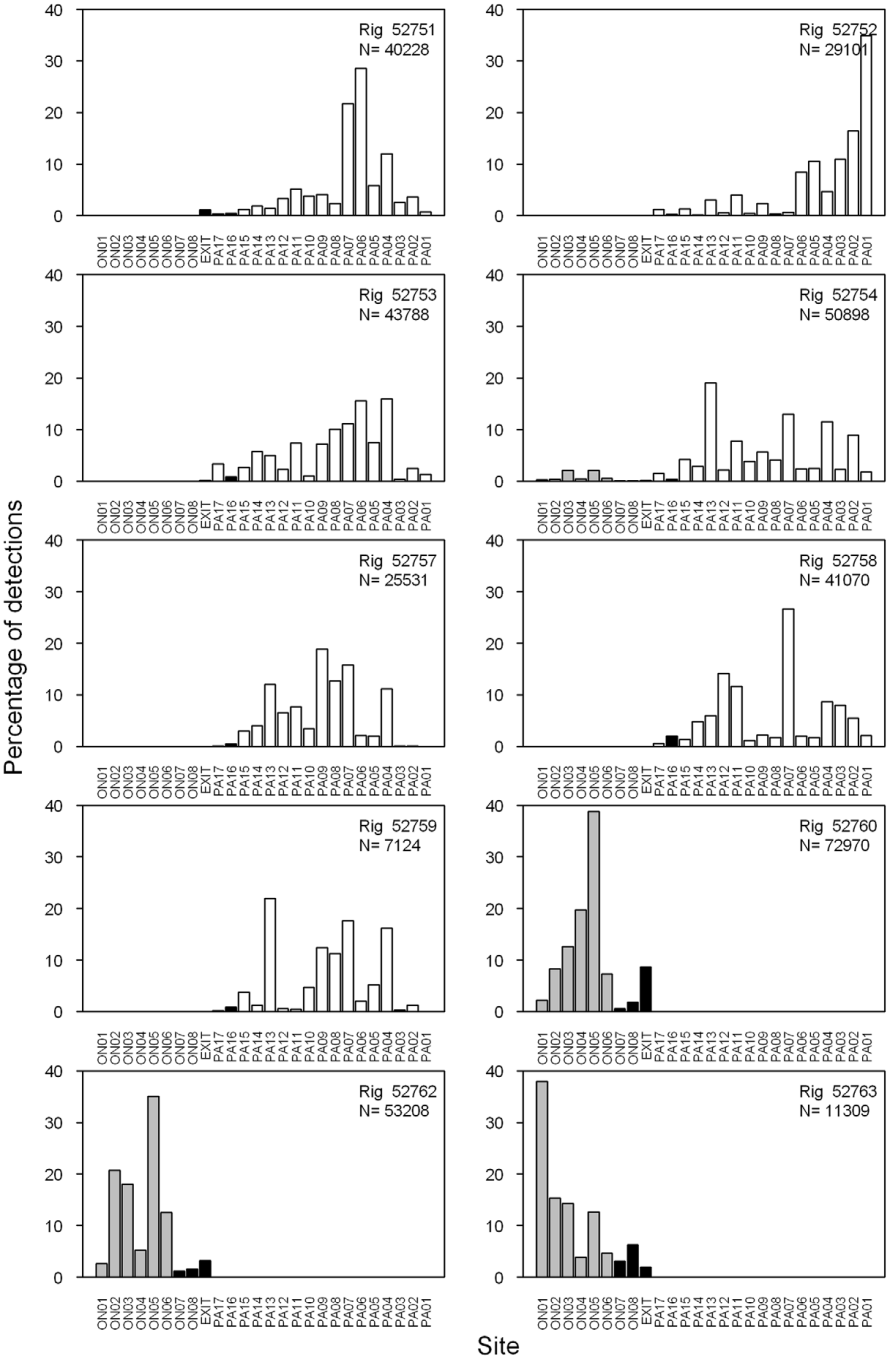
Frequency distribution of acoustic tag detections by site and rig. Sites are shown in Figure 1 and are grouped by Onepoto Arm (grey bars), Pauatahanui Inlet (white), and channels (black). N, sample size.

Nevertheless, inspection of the data revealed that individual rig preferred different parts of the harbour. For example, in Pauatahanui, rig 52751 preferred sites in the south and north (PA04, PA06 and PA07) whereas rig 52752 had a strong preference for the eastern end (PA01 and PA02). In Onepoto, rig 52760 and 52762 were detected most often at the northern end of the basin (ON05) whereas rig 52763 usually occurred at the southern end (ON01) (Figure 4).

**Figure 5:**
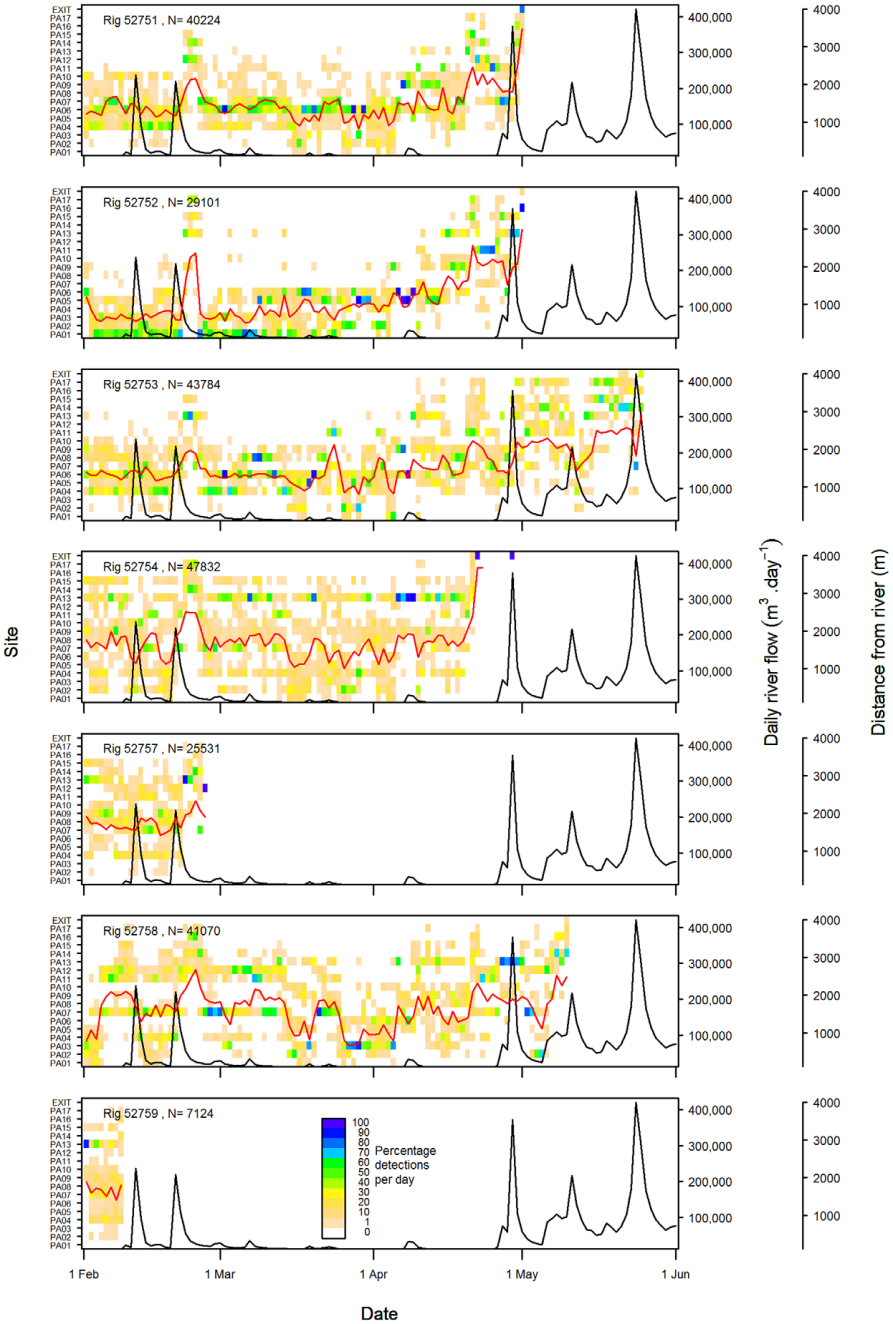
Percentage of acoustic detections recorded per day by site for rig released in Pauatahanui Inlet. Not included are nine days of records for rig 52754 which moved to Onepoto Arm on 21 April (see Figure 6). Also shown are the mean distance from the nearest river (red lines) and daily river flows in Pauatahanui Stream (black lines).

**Figure 6:**
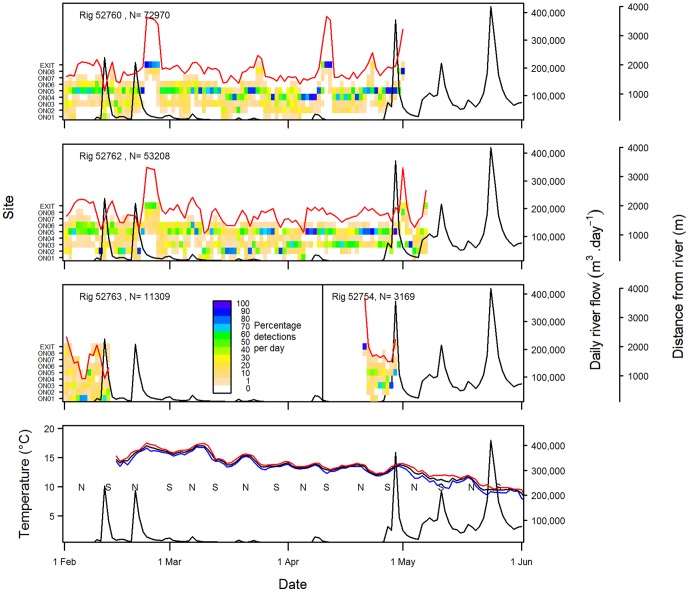
Percentage of acoustic detections recorded per day by site for rig released in Onepoto Arm. Also included (third panel, right) are nine days of records for rig 52754 which was tagged and released in Pauatahanui Inlet and then moved to Onepoto Arm on 21 April (see Figure 5). Also shown are the mean distance from the nearest river (red lines) and daily river flows in Pauatahanui Stream (black lines). The bottom panel compares maximum, mean and minimum daily sea surface temperatures (red, black and blue lines respectively) with river flow rates and tide state (N = neap, S = spring).

One rig (52754) spent time in both arms of Porirua Harbour (Figure 4). It was tagged and released in Pauatahanui, where it remained for 2.6 months before it moved to Onepoto on 21 April for 9 days, and then exited the harbour (Figures 5, 6). The other nine rig remained in the same arm that they were caught and released in until they departed from Porirua Harbour. The PS Index between rig tagged in the two different arms was 0.06 for rig 52754 and 0.001 or less for all other comparisons (all non-significant). This indicates that there is little exchange of juvenile rig between the two harbour arms.

In Pauatahanui, five rig were tracked for more than one month (Figure 5). Rig 52751, 52752 and 52753 spent most of their time in the eastern part of the arm in February and March, but spent increasing amounts of time in the western part in April (and May for rig 52753). Rig 52754 and 52758 ranged throughout Pauatahanui Inlet for the duration of the study, but spent a higher proportion of their time in the western arm in March–April and April–May respectively. In Onepoto, two rig were tracked for more than one month (Figure 6) but they showed no tendency to move towards the channels at the northern end of the arm over time. The single 1+ rig (52763) had a short track record of 13 days and ranged throughout Onepoto arm on most days, but there were insufficient data to determine whether its behaviour differed from that of 0+ rig (Figure 6).

Rig also showed distinct temporary movements towards the outer parts of both arms (western Pauatahanui, northern Onepoto). All rig present in the harbour in late February showed a moderate to strong movement into the outer arms for 3–4 days beginning on 21 February (Figures 5, 6). One rig (52760) also moved from shallow inner sites to deeper outer sites during 6–12 April. From mid April to late May, all rig remaining in Pauatahanui spent much of their time at the outer sites until their departure from the harbour. Such a move was not apparent for the two rig remaining in Onepoto in late April.

The general movement of rig into the outer harbour arms in late February immediately followed a big spike in the daily flow rate of Pauatahanui Stream (Figures 5, 6), and presumably also of other streams draining into both harbour arms. The movement of one rig into outer sites in early April also coincided with a small spike in river flow. Seven out of 10 rig departed from Porirua Harbour during or within a few days of peak river flows – one in mid February, one in late February, four at the end of April, and one in late May (Figures 5, 6). Two other rig (52758, 52762) departed during early May, 7–10 days after a large late-April river spike and during a succeeding moderate flow rate. Rig 52759 departed in mid February before any significant freshwater flow was recorded. Not all river spikes resulted in movement of rig towards the outer harbour: a spike in mid February had no discernible effect on all rig except 52763, which departed from Onepoto the day after the peak river flow.

Despite there being an apparent link between some river spikes and rig distribution, the overall relationship between distance from the nearest river and river flow rate was weak: correlation coefficients were statistically significant for only two of eight rig (rig 52751: p = 0.003; rig 52753: p = 0.000). Lagging the distance time series by 1–6 days led to only one additional significant correlation (rig 52758: p = 0.006 at a lag of 3 days) but made the correlation for rig 52751 non-significant.

SST at the harbour entrance declined steadily during the experiment: the monthly means of the mean daily SSTs from February to May were 17.5, 16.6, 14.7 and 12.0°C. Although there were oscillations of up to 2°C at 7–14 day intervals over this period, there was no obvious link between SST and spikes in river flow rates or tidal state (Figure 6).

**Figure 7:**
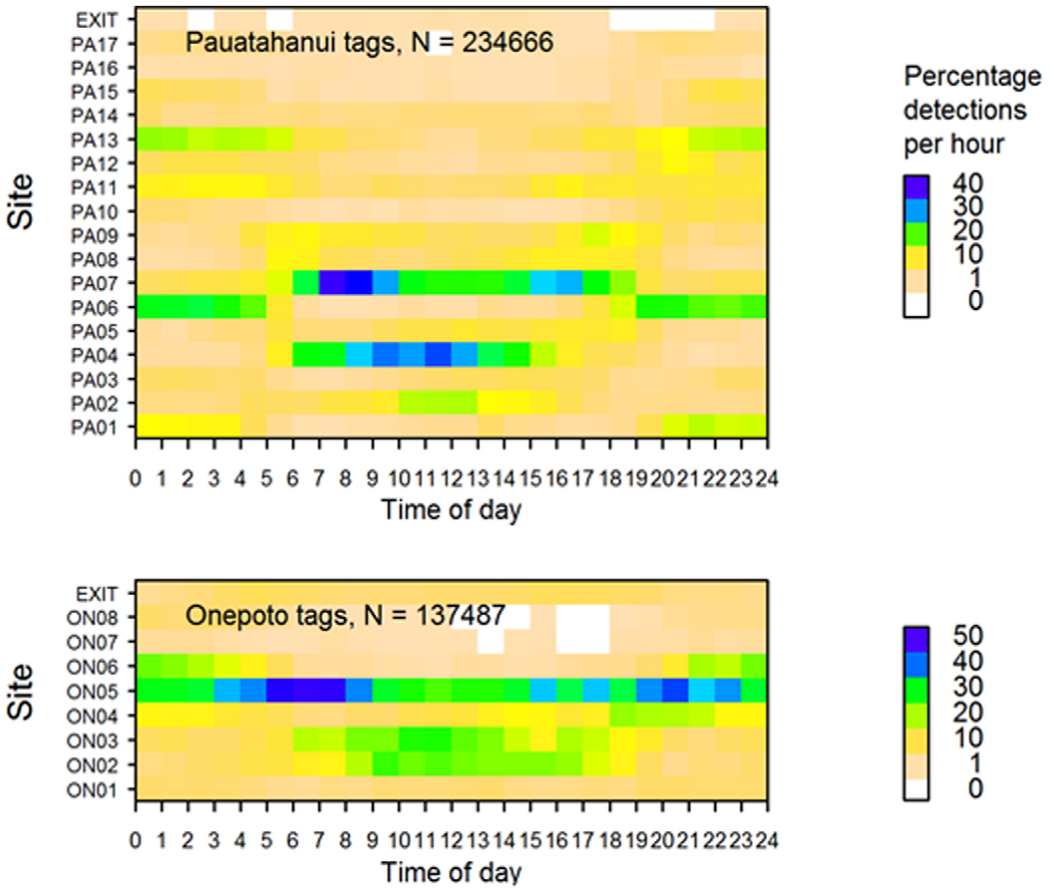
Percentage of acoustic detections recorded per hour by site. Rig released in Pauatahanui Inlet (top) and Onepoto Arm (bottom) (all tags combined). Colour scales differ between the two panels.

**Figure 8:**
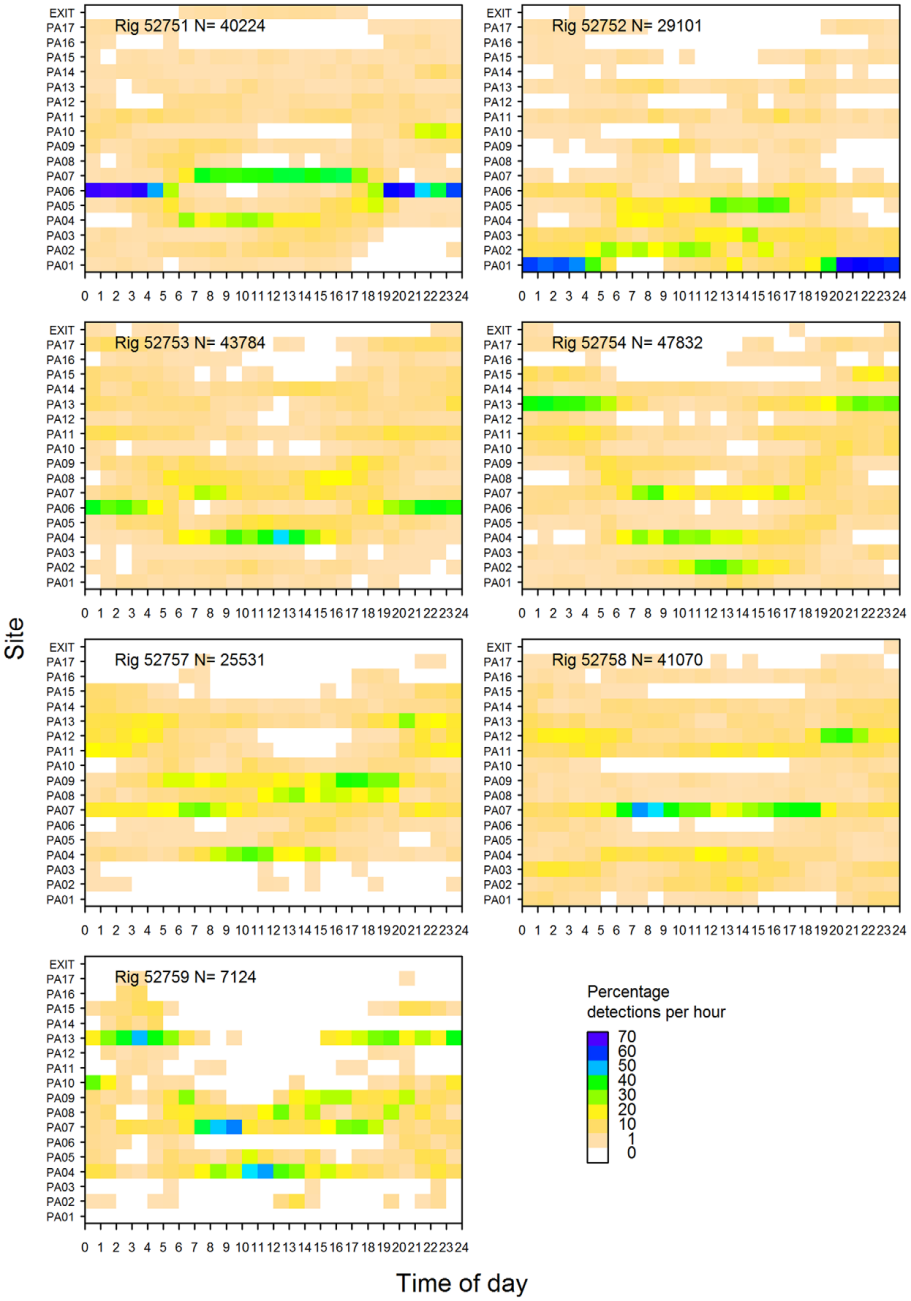
Percentage of acoustic detections recorded per hour by site for rig released in Pauatahanui Inlet.

Rig exhibited strong diel patterns in their use of sites in both Pauatahanui and Onepoto. In Pauatahanui, sites PA01, PA06 and PA13 were inhabited mainly at night, whereas sites PA02, PA04 and PA07 were inhabited mainly during the day (Figure 7). This pattern applied also to individual rig, but each rig showed different site preferences (Figure 8). Sites in the northern and eastern sides of Pauatahanui, which are characterised by very shallow, flat bays, were preferred at night and sites along the southern side, which were in the deeper basin, were preferred during the day. Inspection of the raw data showed that rig travelled daily across the inlet between their preferred day and night sites.

**Figure 9:**
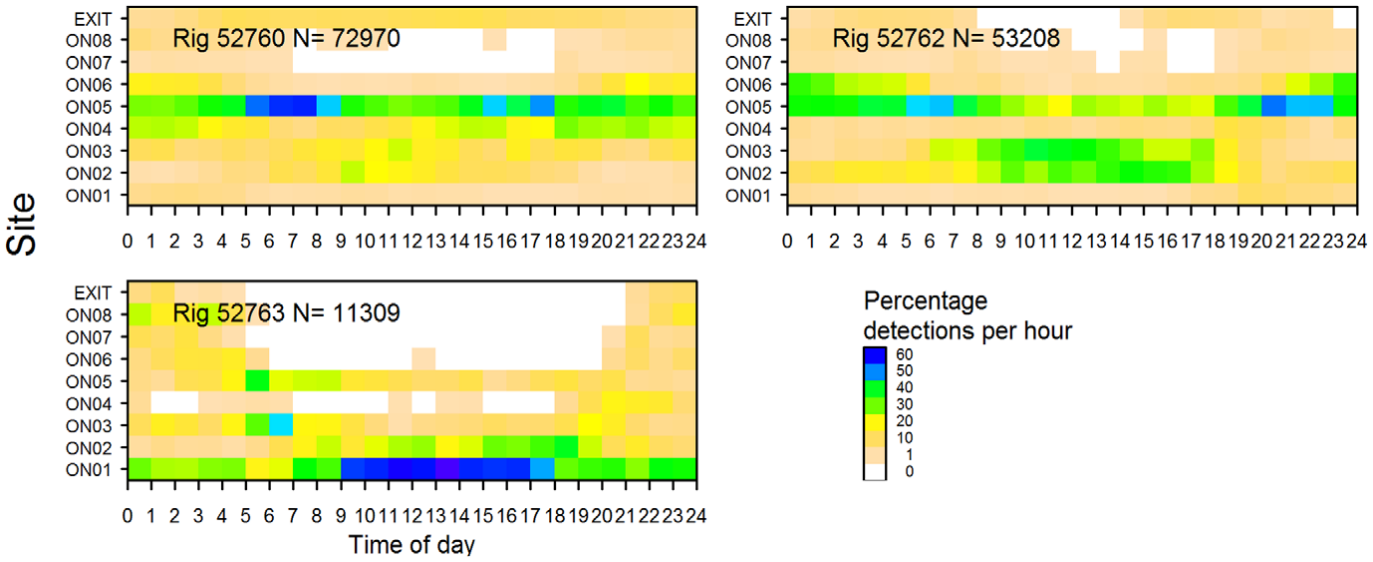
Percentage of acoustic detections recorded per hour by site for rig released in Onepoto Arm.

In Onepoto, site ON05 was occupied at all times of day, although highest detection rates were between 0300–0900 hours and 1500–2300 hours (Figure 7). Sites ON02 and ON03 were used mainly during the day, and site ON06 at night. The three rig in Onepoto had different site preferences and showed some diel differences, though not as clearly as rig in Pauatahanui (Figure 9).

**Figure 10:**
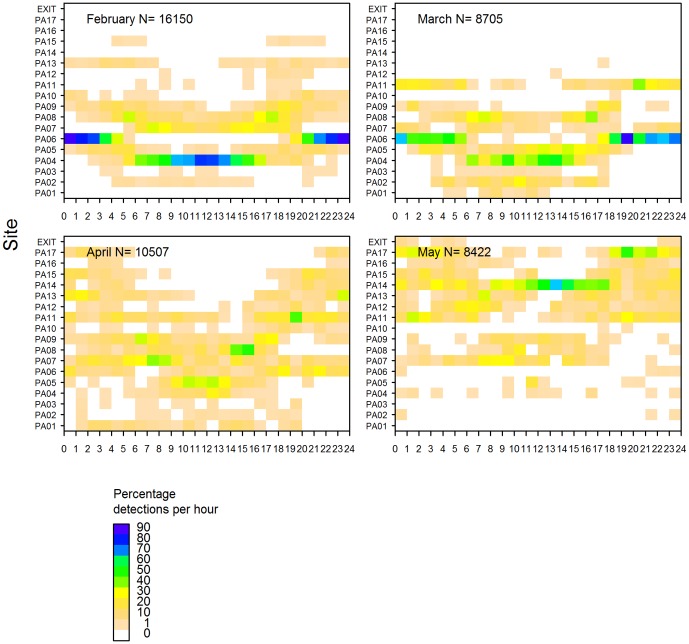
Percentage of acoustic detections by hour, site and month for rig 52753 released in Pauatahanui Inlet. N, sample size.

For most rig, diel variation in site use declined during the experiment. For example, rig 52753 showed very strong diel variation in February, strong variation in March, little variation in April, and weak variation in May (Figure 10). The overall decline in diel migratory behaviour therefore coincided with rig spending more time in the outer parts of the harbour (Figure 5).

## Discussion

This study, in combination with previous work, demonstrates that some large harbours and estuaries in New Zealand satisfy all three of Heupel et al.'s [2] criteria for designation as rig nurseries:

1. Small juveniles occur in high numbers at specific locations throughout North Island. Rig densities are much greater in shallow sheltered parts of several large harbours and estuaries than in deeper parts of those habitats, and other coastal habitats. However, high densities of juvenile rig have not so far been identified from South Island harbours and estuaries despite three nationwide juvenile rig surveys of varying intensities having been conducted in 1985, 2001 and 2009 [33], [35], [36].
2. Juvenile rig remain in these harbours and estuaries from soon after birth for up to seven months. In Porirua Harbour, rig are born in or near the harbour in spring (October to December) and some remain there until late autumn (May) [16], [17], [35].
3. Juvenile rig are repeatedly found in the same locations. Three or more surveys between the 1980s and 2011 have recorded high rig densities in each of Kaipara Harbour, Waitemata Harbour, and Porirua Harbour [16], [17], [33], [35], [37].

Nursery areas may be attractive to juvenile fishes because they provide high abundance and/or density of prey, or because they offer a refuge from predators [38], [39]. Juvenile rig feed intensively during their first few months in estuaries and harbours. A feeding study carried out in Porirua Harbour one year after the present study found that 0+ rig feed mainly on mud crabs [40]. In other rig nurseries, the diet is also dominated by crustaceans, mainly crabs and snapping shrimps. A low proportion of empty stomachs (3%) and high growth rates (1.1–1.5 mm per day) in these nurseries suggest that rig are accessing abundant food resources while there [16], [35], [40].

Rig nurseries occur in estuaries or muddy harbours where water clarity is typically low [33], which may offer protection from visual predators. In Porirua Harbour, potential predators are few and rare. The most likely is the common conger eel (*Conger verreauxi*) which grows to at least 2.2 m TL, is a voracious piscivore, and lives permanently in the estuary, though no information is available on its abundance. Other possible predators that occur at least seasonally in adjacent coastal waters include sevengill shark (*Notorynchus cepedianus*), school shark (*Galeorhinus galeus*), kingfish (*Seriola lalandi*) and bottlenose dolphin (*Tursiops truncatus*). Sevengill sharks have been identified as major predators of *Mustelus antarcticus* in Australia [41], and sevengill sharks and school sharks prey on *Mustelus schmitti* in Argentina [42], [43]. However, none of these fish species has been recorded from Porirua Harbour apart from neonate school sharks [17], [44], which are too small to be a threat to rig; furthermore, bottlenose dolphins are extremely rare visitors (M. P. Francis, unpublished data). Thus predator pressure may be low in the harbour. Consequently, Porirua Harbour may provide both rich food resources and a refuge from predators for young rig.

The acoustic tracking experiment reported here began at the end of January when juvenile rig were deemed large enough to be able to carry acoustic tags. The rig are likely to have been 2–3 months old at that time [16], so the data presented here cover sharks that are about 2–7 months old. Mean rig length was 39 cm TL at the start of the experiment, and these sharks would have grown to about 50 cm in May [16]. Although the sample size in this study was low, there is no reason to believe that the tagged rig were not representative of the population present in Porirua Harbour. Furthermore, the complete spatial coverage of receivers in the harbour mitigates the potential impact of small sample size.

From February to May, rig showed clear site preferences within each harbour arm, but those preferences varied among rig and over time. They spent most of their time in large basins and on shallow sand and mudflats around the margins, and avoided deep channels near the mouth of each arm. Nevertheless, they periodically moved through most of the arm, traversing a straight-line distance of over 3 km. Habitat range increased during autumn (April–May) for many of the Pauatahanui rig: they spent more time in the outer parts of each arm. There was no apparent range increase in Onepoto arm, perhaps because the available habitat area was considerably smaller than in Pauatahanui, and there was no space to expand into other than the channels near the mouth. Absolute habitat ranges could be calculated by estimating “centres of activity” using a mean of the receiver locations weighted by the number of detections and then applying minimum convex polygon or kernel utilisation distribution estimation methods [45], [46]. This was not attempted here because few detections were obtained from multiple receivers, rig can move considerable distances over short time periods, and it was clear from the data that juvenile rig range over an entire harbour arm.

Porirua rig range throughout one harbour arm (rarely two) over the course of several months, occupying a habitat of 2–7 km^2^. Only one out of 10 tagged rig spent time in both Pauatahanui and Onepoto, indicating that there was little movement between the two physically distinct arms. Despite the small sample size, this conclusion is supported by observed differences in the mean lengths of juveniles in the two arms that are maintained throughout the summer–autumn nursery period [35]. Observations of such fine-scale spatial segregation in sharks are uncommon, though a similar situation was reported for blacktip reef sharks (*Carcharhinus melanopterus*) in the lagoon of a coral atoll [25]. The spatial range of rig may be limited by the geography of the harbour – moving further afield would result in their exiting from the harbour, or moving into another harbour arm via deep channels through which strong tidal currents flow. Other important rig nurseries occur in harbours that are much larger (Raglan Harbour 32 km^2^ and Kaipara Harbour 743 km^2^) than Porirua Harbour (7 km^2^). Set net surveys of Raglan Harbour and two arms of the very large Kaipara Harbour indicate that, as in Porirua, rig are most abundant in the upper, shallow, turbid parts of each [33]. An important next step is to define the spatial distribution of rig nurseries within larger harbours, and determine whether individual rig roam more widely when they are not physically constrained by harbour limits or unsuitable habitat.

Rig moved to outer sites, including sites where receivers were deployed in channels, following some spikes in river flow rates; most rig also left the harbour permanently during or soon after a river spike. This suggests that rig avoid low salinity water by moving into deeper channels where a saltwater wedge is probably present (salinity stratification has been observed at the entrance to Porirua Harbour after high rainfall [24]). Jones & Hadfield [17] also found by set net sampling that juvenile rig moved into the deeper channels of Porirua Harbour in April before leaving the estuary in late autumn.

Other factors may also be involved in the displacement of rig towards the outer part of the harbour arms. The first river flow spike during this study (in mid February) may have precipitated the departure of one tagged rig, but all other sharks showed no response to it; by contrast a similar peak nine days later was followed by a general movement by all rig in both arms towards the harbour mouth. Freshwater inflow may need to reach a cumulative threshold before rig are displaced seawards. Alternatively, the salinity regime in the harbour may be modified by tidal state: the first spike occurred during a spring tidal period whereas the second spike occurred during a neap tidal period (Figure 6). Spring tides may inject sufficient seawater into the harbour during flood tides to ameliorate the effect of the freshwater inflow; however spring tides should equally result in a greater loss of high salinity water during ebb tides. Furthermore, other river flow spikes that displaced rig seawards also coincided with spring tides (Figures 5, 6).

Juvenile rig behaviour observed in this study was similar to that reported for a number of other shark species that live in shallow coastal nurseries. Expansion of home ranges with increasing body size is common but not universal in juvenile sharks [47]. Blacktip sharks (*Carcharhinus limbatus*) showed a dramatic and near-synchronous increase in home range in July in two consecutive years, though the reason for this was not clear [4]. Horizontal shifts in home ranges without accompanying range expansion have also been reported for bonnethead sharks *Sphyrna tiburo* and pigeye sharks *Carcharhinus amboinensis* [5], [48]. Bull sharks *Carcharhinus leucas* prefer low salinities (7.5–17.5 psu), but will leave estuaries for the open coast if salinity drops too low during floods [49]. High riverine inflows to a coastal bay in Australia displaced pigeye sharks to less affected areas [48]. Blacktip sharks were also observed to leave a shallow bay and move into deeper water in advance of an approaching tropical storm, possibly in response to dropping barometric pressure [50].

Juvenile rig showed strong diel movements in Pauatahanui during February–March, although the diel pattern weakened in April–May. Weaker diel site preferences were also observed in Onepoto. Preferred Pauatahanui day and night habitats differed in depth (deeper and shallower respectively) and probably also other features, but no measurements were made of their physical characteristics. Persistent use of the same day and night sites indicates that diel movements are directed rather than random. Young lemon sharks, *Negaprion brevirostris*, can return to their home ranges when displaced 4–16 km, confirming the existence of precise navigational ability [51].

Diel movement patterns might reflect diel feeding patterns, with rig moving from day resting sites along the southern side of Pauatahanui to night feeding sites in the northern and eastern parts of the arm. Getzlaff [40] did not detect any diel differences in juvenile rig feeding intensity, and found that most stomachs contained food regardless of the time of day. However, her stomach samples were not collected from specific time periods and she did not determine the state of digestion of the food or the prey evacuation rate, so her data were not adequate for addressing this question. Similarly, juvenile smooth dogfish *Mustelus canis* show little diel variation in stomach fullness [52]. Juvenile smooth dogfish and gray smoothhound *M. californicus* show greater swimming activity at night [45], [53], and diel variation in habitat has been reported in smooth dogfish, gray smoothhound, gummy shark (*Mustelus antarcticus*) and brown smoothhound *M. henlei* [45], [52], [54], [55]. Increased feeding at night has been reported in juvenile scalloped hammerhead sharks (*Sphyrna lewini*) [56]. Other shark species, including sandbar shark *Carcharhinus plumbeus*, blacktip shark and leopard shark *Triakis semifasciata*, exhibit diel activity patterns varying from strong to negligible [47], [57]–[59]. However, it remains unknown whether increased night activity in *Mustelus* species is a result of them feeding mainly at night. Further work is required to determine whether the diel movements observed for juvenile rig are related to a feeding cycle.

Tides also influence activity patterns in gray and brown smoothhounds [45], [54], and it is possible that tide state modifies the diel movements of rig. However such an effect is unlikely to be major, as a strong tidal response would obliterate any diel cycle.

Estuarine harbours around North Island, New Zealand, are used as nurseries by neonate and juvenile rig up to an age of about 7 months, after which they move out to open coastal waters and the continental shelf. The presence of rig in shallow coastal habitats during their early life exposes them to a range of human stressors, particularly activities that modify and degrade their habitat [60]. Juvenile rig may also be taken as bycatch in small mesh nets (mainly 81–100 mm stretched mesh) set for grey mullet (*Mugil cephalus*) and yellow-eyed mullet (*Aldrichetta forsteri*) [60], but rig are not vulnerable to line-fishing as they seldom take hooks unless they are baited with crustaceans.

Elsewhere, a range of charcharhinid, hexanchid and sphyrnid shark species have small home ranges and strong site fidelity, particularly as juveniles [4]–[11]. Acoustic telemetry studies have provided considerable insight into the scale of MPAs required, and the range of habitat types that need to be incorporated in them, to adequately protect species and life history stages with varying home ranges [6], [58]. Simulations and other analyses suggest that MPAs of only a few square kilometres can provide significant protection to species with a high degree of site fidelity, although inevitably large reserves are likely to provide more protection than small reserves, particularly for older age classes whose home ranges expand with age [7], [9], [12].

Protection of nursery areas has not proven to be adequate for managing shark stocks in isolation, but MPAs can effectively complement other management measures applied to older age groups [6], [61]. Rig fisheries are managed under a Quota Management System by application of restrictive Total Allowable Catches [14]. MPAs offer the potential to protect rig nursery grounds from direct physical impacts such as dredging and dumping of spoil, reclamation and marina construction, and also from indirect fishing mortality, thus enhancing recruitment of juveniles to the adult population. MPAs will also protect other fish species that use them as nursery grounds, such as juvenile snapper (*Pagrus auratus*) and grey mullet [33].

The overall goal of this study was to estimate the size of MPA required to protect juvenile rig on their estuarine nursery grounds. This study has shown that rig range over the entire Pauatahanui arm (4 km^2^) and occasionally both arms of Porirua Harbour (7 km^2^), suggesting that an effective MPA would need to cover the entire Porirua Harbour. For other larger harbours, 7 km^2^ is likely to be a lower bound on the size required. Further research is required to determine the sizes of rig home ranges in harbours where nursery habitat is much more extensive and rig are free to roam more widely than in Porirua Harbour. Furthermore, quantification of the number of recruits generated by known rig nurseries [33], and determination of which parent stock each nursery supplies, are important pre-requisites for designing a network of estuarine MPAs with the aim of maintaining recruitment to adult rig populations.

MPAs do not provide a mechanism for controlling land-based impacts such as accelerated sedimentation and heavy metal pollution. Integration of marine and terrestrial management tools across a range of central and local government agencies and stakeholders will be essential to fully protect rig nursery areas from degradation or loss.

## References

[1] Beck MW, Heck KL, Able KW, Childers DL, Eggleston DB, et al (2001) The identification, conservation and management of estuarine and marine nurseries for fish and invertebrates. Bioscience 51: 633–641.

[2] Heupel MR, Carlson JK, Simpfendorfer CA (2007) Shark nursery areas: concepts, definition, characterization and assumptions. Mar Ecol Prog Ser 337: 287–297.

[3] Ministry of Fisheries (2008) New Zealand national plan of action for the conservation and management of sharks. Wellington: Ministry of Fisheries. 90 p.

[4] Heupel MR, Simpfendorfer CA, Hueter RE (2004) Estimation of shark home ranges using passive monitoring techniques. Env Biol Fish 71: 135–142.

[5] Heupel MR, Simpfendorfer CA, Collins AB, Tyminski JP (2006) Residency and movement patterns of bonnethead sharks, *Sphyrna tiburo*, in a large Florida estuary. Env Biol Fish 76: 47–67.

[6] Chapman DD, Pikitch EK, Babcock EA, Shivji MS (2005) Marine reserve design and evaluation using automated acoustic telemetry: a case-study involving coral reef-associated sharks in the Mesoamerican Caribbean. Mar Tech Soc J 39: 42–55.

[7] Garla RC, Chapman DD, Wetherbee BM, Shivji MS (2006) Movement patterns of young Caribbean reef sharks, *Carcharhinus perezi*, at Fernando de Noronha Archipelago, Brazil: the potential of marine protected areas for conservation of a nursery ground. Mar Biol 149: 189–199.

[8] Field IC, Meekan MG, Speed CW, White W, Bradshaw CJA (2011) Quantifying movement patterns for shark conservation at remote coral atolls in the Indian Ocean. Coral Reefs 30: 61–71.

[9] Barnett A, Abrantes KG, Stevens JD, Semmens JM (2011) Site fidelity and sex-specific migration in a mobile apex predator: implications for conservation and ecosystem dynamics. Anim Behav 81: 1039–1048.

[10] Barnett A, Abrantes KG, Seymour J, Fitzpatrick R (2012) Residency and spatial use by reef sharks of an isolated seamount and its implications for conservation. PLoS ONE 7: e36574. 22615782 10.1371/journal.pone.0036574 PMC3353940

[11] Bond ME, Babcock EA, Pikitch EK, Abercrombie DL, Lamb NF, et al (2012) Reef sharks exhibit site-fidelity and higher relative abundance in marine reserves on the Mesoamerican Barrier Reef. PLoS ONE 7: e32983. 22412965 10.1371/journal.pone.0032983 PMC3297616

[12] Heupel MR, Simpfendorfer CA (2005) Using acoustic monitoring to evaluate MPAs for shark nursery areas: the importance of long-term data. Mar Tech Soc J 39: 10–18.

[13] Knip DM, Heupel MR, Simpfendorfer CA (2010) Sharks in nearshore environments: models, importance, and consequences. Mar Ecol Prog Ser 402: 1–11.

[14] Ministry of Fisheries Science Group (2011) Report from the mid-year Fisheries Assessment Plenary, November 2011: stock assessments and yield estimates. Unpublished report held in NIWA library, Wellington. 355 p.

[15] Francis MP, Mace JT (1980) Reproductive biology of *Mustelus lenticulatus* from Kaikoura and Nelson. N Z J Mar Freshwater Res 14: 303–311.

[16] Francis MP, Francis RICC (1992) Growth rate estimates for New Zealand rig (*Mustelus lenticulatus*). Aust J Mar Freshwater Res 43: 1157–1176.

[17] Jones JB, Hadfield JD (1985) Fishes from Porirua and Pauatahanui Inlets: occurrence in gill nets. N Z J Mar Freshwater Res 19: 477–484.

[18] King KJ (1984) Changes in condition of mature female rig (*Mustelus lenticulatus*) from Golden Bay in relation to seasonal inshore migrations. N Z J Mar Freshwater Res 18: 21–27.

[19] Blaschke P, Woods J, Forsyth F (2010) The Porirua Harbour and its catchment: a literature summary and review. Report for Porirua City Council. 99 p.

[20] Gibb JG, Cox GJ (2009) Patterns and rates of sedimentation in Porirua Harbour. Report for Porirua City Council. 65 p.

[21] Irwin J (1978) Pauatahanui Inlet bathymetry 1∶5000. N Z Oceanogr Inst Chart Misc Ser

[22] Irwin J (1978) Porirua Inlet bathymetry 1∶5000. N Z Oceanogr Inst Chart Misc Ser

[23] Discovery Marine (2009) Porirua Harbour survey. Report of survey. Report prepared for Porirua City Council. 32 p.

[24] Förch EC (1983) Studies on the zooplankton of Pauatahanui Inlet [PhD thesis]. Wellington, New Zealand: Victoria University. 192 p.

[25] Papastamatiou YP, Friedlander AM, Caselle JE, Lowe CG (2010) Long-term movement patterns and trophic ecology of blacktip reef sharks (*Carcharhinus melanopterus*) at Palmyra Atoll. J Exp Mar Biol Ecol 386: 94–102.

[26] Meyer CG, Clark TB, Papastamatiou YP, Whitney NM, Holland KN (2009) Long-term movement patterns of tiger sharks *Galeocerdo cuvier* in Hawaii. Mar Ecol Prog Ser 381: 223–235.

[27] Linton LR, Davies RW, Wrona FJ (1981) Resource utilization indices: an assessment. J Anim Ecol 50: 283–292.

[28] Feinsinger P, Spears EE, Poole RW (1981) A simple measure of niche breadth. Ecol 62: 27–32.

[29] Ricklefs RE, Lau M (1980) Bias and dispersion of overlap indices: results of some Monte Carlo simulations. Ecol 61: 1019–1024.

[30] Gotelli NJ, Graves GR (1996) Null models in ecology. Washington, DC: Smithsonian Institution Press.

[31] Gotelli NJ, Entsminger GL (2012) EcoSim 7.72. Acquired Intelligence, Inc.

[32] Sokal RR, Rohlf FJ (1981) Biometry. The principles and practice of statistics in biological research. New York: Freeman. 859 p.

[33] Francis M, Lyon W, Jones E, Notman P, Parkinson D, et al.. (2012) Rig nursery grounds in New Zealand: a review and survey. N Z Aquat Envt Biodiv Rep. 50 p.

[34] Bridger CJ, Booth RK (2003) The effects of biotelemetry transmitter presence and attachment procedures of fish physiology and behavior. Rev Fish Sci 11: 13–34.

[35] Hendry RT (2004) An assessment of the spatial extent and relative importance of nurseries, and of the genetic structure among nurseries of rig (*Mustelus lenticulatus*), an endemic New Zealand shark [MSc]. Wellington: Victoria University of Wellington. 210 p.

[36] Hurst RJ, Bagley NW, Anderson OF, Francis MP, Griggs LH, et al.. (2000) Atlas of juvenile and adult fish and squid distributions from bottom and midwater trawls and tuna longlines in New Zealand waters. NIWA Tech Rep. 162 p.

[37] Briggs I (1980) Upper Waitemata Harbour - interim fish survey. Upper Waitemata Harbour Catchment Study Working Rep. 28 p.

[38] Simpfendorfer CA, Milward NE (1993) Utilisation of a tropical bay as a nursery area by sharks of the families Carcharhinidae and Sphyrnidae. Env Biol Fish 37: 337–345.

[39] Heupel MR, Hueter RE (2002) Importance of prey density in relation to the movement patterns of juvenile blacktip sharks (*Carcharhinus limbatus*) within a coastal nursery area. Mar Freshwater Res 53: 543–550.

[40] Getzlaff C (2012) Diet and foraging behaviour of juvenile rig (*Mustelus lenticulatus*) from New Zealand harbours and estuaries [M.Sc. thesis]. Palmerston North: Massey University. 102 p.

[41] Barnett A, Abrantes KG, Stevens JD, Bruce BD, Semmens JM (2010) Fine-scale movements of the broadnose sevengill shark and its main prey, the gummy shark. PLoS ONE 5: 1–10. 10.1371/journal.pone.0015464 PMC2997065 21151925

[42] Lucifora LO, Menni RC, Escalante AH (2005) Reproduction, abundance and feeding habits of the broadnose sevengill shark *Notorynchus cepedianus* in north Patagonia, Argentina. Mar Ecol Prog Ser 289: 237–244.

[43] Lucifora LO, García VB, Menni RC, Escalante AH (2006) Food habits, selectivity, and foraging modes of the school shark *Galeorhinus galeus* . Mar Ecol Prog Ser 315: 259–270.

[44] Healy WB (1980) Pauatahanui Inlet - an environmental study. DSIR Inf Ser. 198 p.

[45] Espinoza M, Farrugia TJ, Lowe CG (2011) Habitat use, movements and site fidelity of the gray smooth-hound shark (*Mustelus californicus* Gill 1863) in a newly restored southern California estuary. J Exp Mar Biol Ecol 401: 63–74.

[46] Simpfendorfer CA, Heupel MR, Hueter RE (2002) Estimation of short-term centers of activity from an array of omnidirectional hydrophones and its use in studying animal movements. Can J Fish Aquat Sci 59: 23–32.

[47] Speed CW, Field IC, Meekan MG, Bradshaw CJA (2010) Complexities of coastal shark movements and their implications for management. Mar Ecol Prog Ser 408: 275–293.

[48] Knip DM, Heupel MR, Simpfendorfer CA, Tobin AJ, Moloney J (2011) Wet-season effects on the distribution of juvenile pigeye sharks, *Carcharhinus amboinensis*, in tropical nearshore waters. Mar Freshwater Res 62: 658–667.

[49] Simpfendorfer CA, Freitas GG, Wiley TR, Heupel MR (2005) Distribution and habitat partitioning of immature bull sharks (*Carhcarhinus leucas*) in a southwest Florida estuary. Estuaries 28: 78–85.

[50] Heupel MR, Simpfendorfer CA, Hueter RE (2003) Running before the storm: blacktip sharks respond to falling barometric pressure associated with tropical storm Gabrielle. J Fish Biol 63: 1357–1363.

[51] Clermont S, Gruber SH (2005) Homing ability of young lemon sharks, *Negaprion brevirostris* . Env Biol Fish 72: 267–281.

[52] Rountree RA, Able KW (1996) Seasonal abundance, growth, and foraging habits of juvenile smooth dogfish, *Mustelus canis*, in a New Jersey estuary. Fish Bull 94: 522–534.

[53] Casterlin ME, Reynolds WW (1979) Diel activity patterns of the smooth dogfish shark, *Mustelus canis* . Bull Mar Sci 29: 440–442.

[54] Campos BR, Fish MA, Jones G, Riley RW, Allen PJ, et al (2009) Movements of brown smoothhounds, *Mustelus henlei*, in Tomales Bay, California. Env Biol Fish 85: 3–13.

[55] Barnett A, Semmens JM (2012) Sequential movement into coastal habitats and high spatial overlap of predator and prey suggest high predation pressure in protected areas. Oikos 121: 882–890.

[56] Bush A (2003) Diet and diel feeding periodicity of juvenile scalloped hammerhead sharks, *Sphyrna lewini*, in Kane'ohe Bay, O'ahu, Hawai'i. Env Biol Fish 67: 1–11.

[57] Rechisky EL, Wetherbee BM (2003) Short-term movements of juvenile and neonate sandbar sharks, *Carcharhinus plumbeus*, on their nursery grounds in Delaware Bay. Env Biol Fish 68: 113–128.

[58] Heupel MR, Simpfendorfer CA (2005) Quantitative analysis of aggregation behavior in juvenile blacktip sharks. Mar Biol 147: 1239–1249.

[59] Ackerman JT, Kondratieff MC, Matern SA, Cech JJ (2000) Tidal influences on spatial dynamics of leopard sharks, *Triakis semifasciata*, in Tomales Bay, California. Env Biol Fish 58: 33–43.

[60] Jones E, Francis M, Paterson C, Rush N (in press) Habitats of particular significance for fisheries management: identification of threats and stressors to rig nursery areas. N Z Aquat Envt Biodiv Rep

[61] Kinney MJ, Simpfendorfer CA (2009) Reassessing the value of nursery areas to shark conservation and management. Cons Lett 2: 53–60.

